# Feasibility and Clinical Usefulness of the Unified Protocol in Online Group Format for Bariatric Surgery Candidates: Study Protocol for a Multiple Baseline Experimental Design

**DOI:** 10.3390/ijerph17176155

**Published:** 2020-08-25

**Authors:** Alba Quilez-Orden, Vanesa Ferreres-Galán, Jorge Osma

**Affiliations:** 1 Department of Psychology and Sociology, University of Zaragoza, 44003 Teruel, Spain; aquilez@iisaragon.es; 2 Health Research Institute of Aragón, 50009 Zaragoza, Spain; 3 Mental Health Unit of the Regional Hospital of Vinaròs, 12500 Castellón, Spain; ferreres_van@gva.es

**Keywords:** emotional disorders, transdiagnostic, online group format, unified protocol, bariatric surgery, obesity

## Abstract

Obesity is currently becoming a serious global public health problem due to its high prevalence and continuous increase. This condition is associated with different physical and mental health problems. The presence of emotional disorders (anxiety, depression and related disorders) among candidates for bariatric surgery is very high and predicts worse physical and psychological results. The present study aims to explore the feasibility and clinical usefulness of the Unified Protocol, a transdiagnostic emotion regulation-based intervention, delivered in an online group format to patients with emotional disorder diagnosis or symptoms, who are waiting for bariatric surgery. We will conduct a pilot study with a repeated single-case experimental design (multiple baseline design) in a public mental health service. The sample will consist of 60 participants, who will be randomized to three baseline conditions: 8, 12 or 15 evaluation days before the intervention. Diagnostic criteria, symptomatology and body mass index are the primary outcome measures, and we will include affectivity, personality, quality of life, body image, eating behavior and surgical complications like secondary measures. An analysis of treatment satisfaction will be also performed. Assessment points will include pre-treatment, baseline, treatment, post-treatment, and follow-ups every three months until two years after post-treatment. The results obtained in this study may have important clinical, social and economic implications for public mental health.

## 1. Introduction

Obesity is a chronic disease characterised by an increase in body fatness, which is usually estimated by body mass index (BMI) calculated as measured body weight (kg) divided by measured height squared (m^2^) [1]. Obesity is currently becoming a serious global public health problem due to its high prevalence and increase in recent years [2]. In Spain alone, 25% of the population is obese or overweight, and the expectations are alarming. It is expected that in 2030, up to 80% of Spanish men and 55% of women will be obese or overweight, exceeding 27.2 million of people [3].

The causes of obesity are complex and multifactorial [1], and this condition is associated with different health problems, such as myocardial infarction, hypertension, stroke, dyslipidemia, diabetes mellitus, or obstructive sleep apnea [4,5,6]. All these alterations affect the quality of life and can disturb important areas of functioning (e.g., physical function, vitality, social functioning and emotional role) [7,8]. In addition to all of these complications, people with obesity experience significant psychological difficulties [7]. Due to all of these health implications, obesity represents a challenge to the countries’ economies [9]. As of 2016 in Spain, the obesity cost meant almost two billion euros of extra expenses for the National Health System [3].

For all these reasons (high prevalence and associated costs), many efforts have been made to find treatments for obesity and weight loss, which include lifestyle interventions, pharmacological interventions, surgical interventions, and endoscopic bariatric procedures [7]. Bariatric surgery (BS) is the most commonly performed procedure worldwide [10] and the most effective intervention for individuals with severe obesity (BMI greater than 40 kg/m^2^) [11], and includes a group of surgical procedures performed to facilitate weight loss, such as open or laparoscopic roux-en-Y gastric bypass, sleeve gastrectomy, and adjustable gastric banding. The most obvious and studied benefits of BS refer to physical issues, specifically weight loss and improvement of obesity-related physical comorbidities; however, between 10% and 25% of patients who undergo BS show suboptimal weight loss, and it is estimated that they regain approximately 10% of their weight during the first decade after the intervention [12]. Regarding the psychological benefits of BS, limited and inconclusive results have been found [13]. Some systematic reviews have reported improvements after BS, especially in depressive symptoms, but also limited only to the first months after the intervention, and they tend to reappear after the first two years [10].

Some studies have questioned whether previous psychopathology history could influence the results obtained by BS [2]. This topic is especially relevant because the percentage of psychopathology among people waiting for BS (between 20.9% and 55.5%) [12] is much higher than in the general population (between 12.53% and 13.87%) [14]. Specifically, the group of emotional disorders (ED), which includes anxiety and mood disorders, eating disorders, and related disorders ([15]; Supplementary Materials), are the most prevalent disorders among BS candidates: eating disorders (50%), mood disorders (31.5%), anxiety disorders (24%), and substance use related disorders (10%) [12]. It has been concluded that the presence of EDs among candidates for BS predicts worse results in the long term [11], furthermore, a meta-analysis states that the prevalence of suicide mortality is up to 1.8%, and the prevalence of suicide is up to 0.3% after bariatric surgery [16].

As we can observe, psychological evaluation and intervention play a fundamental role in the multidisciplinary work performed with these patients before and after BS although, until now, it has not received much attention [12]. Improvements in eating psychopathology (e.g., binge eating, emotional eating, body image dissatisfaction) have been reported from psychological interventions using psychoeducation, goal setting, self-monitoring, normalized eating, stimulus control, cognitive restructuring, and relapse prevention [11]. Cognitive–behavioral therapy (CBT) is the one that accumulates the most evidence of the improvement of eating behaviors (e.g., binge eating and emotional eating) and psychological functioning (e.g., quality of life, depression, and anxiety symptoms) [11]. Despite the good outcomes achieved, these CBT interventions have been developed to treat specific disorders (e.g., eating disorders or anxiety disorders), and this fact raises some limitations. First, clinicians’ great effort to specialize in a range of different interventions; second, the increased costs for health systems and clinicians in training, and as a consequence, the difficulties in dissemination and implementation of the CBT interventions [17]. Furthermore, if we consider that the most prevalent disorders among patients with obesity are EDs [12], there are additional limitations to specific CBT interventions. This is because in the case of these disorders, comorbidity is very high, and subclinical symptoms or unspecified disorders usually arise [18]. These comorbid conditions make it even more difficult to choose the best specific CBT treatment in each case [19].

To address all the limitations of the specific CBT treatments previously discussed, in recent years, different CBT interventions from a transdiagnostic approach have been developed and tested. In this sense, the Unified Protocol for the transdiagnostic treatment of EDs (UP) is a CBT emotion-based intervention designed to treat the etiological and maintenance mechanisms shared by all EDs [20]. This intervention focuses on a wide range of emotional psychopathology, considering comorbid disorders and subclinical or unspecified symptoms, reducing treatment times and costs, and improving response to treatment [17].

The UP is made up of eight treatment modules, five of which are considered core modules because they are focused on training different specific emotion-regulation skills [21]. Despite that the UP is a protocolized intervention, it is also flexible and versatile, allowing clinicians to use some of the modules or to change modules’ order to personalize the UP to their patients [22]. Its main objective is to enhance emotion-regulation strategies to all people presenting emotional dysregulation problems [15]. To date, the UP has shown a significant improvement in pre-treatment symptoms and has obtained effect sizes that are at least comparable to existing specific CBT interventions, in on-site and online formats [23]. Beyond the mood or anxiety disorders per se, the UP has been applied to different health problems, such as cancer [24], HIV [25], or irritable bowel syndrome [26]. Despite the positive data on the effectiveness of the UP for the treatment of EDs in different health conditions, we have found no studies about its clinical utility to improve anxiety and depressive symptoms or EDs in patients with obesity who are waiting for BS.

As mentioned, the UP can be applied in various formats, such as onsite or online (both individual and group). Regarding online interventions in mental health (e-Health), there is an important amount of evidence over the last 15 years informing that online treatments are effective to treat a wide range of mental health disorders and that they can be as effective as onsite treatments [27]. We also know that online interventions do not negatively affect the therapeutic relationship [28] and that the users generally show high levels of acceptance and satisfaction with this delivery format [29]. The use of Information and Communication Technologies (ICTs) reduces the burden on health professionals and users, facilitating data collection and accessibility to Treatment [30]. In fact, different studies have shown the need to develop technology-based interventions to increase accessibility to treatment for BS candidates because their obesity condition significantly affects their mobility, and that makes it difficult for them to attend weekly sessions on-site [31]. Another practical benefit of delivering online psychological interventions is related to the current situation caused by the Covid-19 pandemic because the online format (e.g., emails, videoconference, etc.) will allow phycologists to continue their interventions and/or the follow-up assessments in case of new mobility restrictions [32]. Finally, the online group format can offer additional advantages to the online format itself, as it facilitates social support among the participants in the therapy and also allows them to share experiences and learn from each other [33].

Therefore, the general objective of this study is to analyze the feasibility and clinical usefulness of the UP, applied in an online group format, in a mental health setting of the National Health System to candidates for BS who have at least one diagnosis of ED or emotional symptoms.

The specific objectives pursued are: to evaluate adherence to treatment and clinical improvement in the primary and secondary measures after applying the UP and to study its long term clinical usefulness until two years after the intervention. Thus, the main hypotheses proposed for this study are: (1) statistically significant differences (reliable change index; RCI) will be obtained between the scores obtained at the pre-treatment, baseline, and post-treatment on the primary and secondary measures; (2) the improvements obtained after the application of the UP will be maintained in the long term (follow-ups of up to two years); (3) participants in the pilot study will report high adherence rates and high satisfaction scores regarding the treatment delivery format and its components.

## 2. Materials and Methods

This is a pilot study using a repeated single-case experimental design (multiple baseline design) to explore the feasibility and clinical usefulness of a transdiagnostic emotion-based online group intervention (UP) for BS candidates with EDs or subclinical anxious or depressive symptoms attended to in a public mental health service. We have chosen this design for three reasons: (1) the unit of intervention and unit of data analysis is an individual case, specifically a cluster of participants; (2) the case provides its own control for comparison purposes because a number of variables are measured before the intervention and compared with measures during and after the intervention and (3) the variable is repeatedly measured within and across different conditions or levels of the independent variable. Furthermore, and because our main objective is to answer the question “Is the Unified Protocol able to improve the emotional state of people waiting for BS?”, we chose a multiple baseline design to improve experimental control throughout replication, introducing the independent variable at different points in time. The fundamental idea of choosing this type of design is that each of the participants can be their own control group [34].

The multiple baseline design involves the application of the treatment variable in a staggered way over time, through different observational units. This design is suitable for health services research interventions that are focused on changing patient behavior. This methodology facilitates a systematic comparison of pre-intervention and post-intervention measures [35], and conducting a preliminary assessment of a novel intervention [36]. Like randomized controlled trials (RCTs), the multiple baseline design can demonstrate that a significant change in behaviour has occurred and that is result of the intervention. One of the main limitations is that each participant must show changes only when the intervention is applied, and this issue, at a practical level is complicated, can make it difficult for the researcher to draw clear conclusions about the impact of the intervention. In order to reduce this limitation and improve the internal validity, researchers start treatment at different times across settings, behaviors, or people [37]. The multiple baseline design has advantages over the RCTs because this design requires fewer population groups and communities and they may act as their own controls [35]. In this sense, researchers often use this design with several people at once addressing the issue of external validity [37].

In the present investigation, all consecutive patients who are selected to undergo BS and who present anxiety or depressive symptoms or at least one diagnosis of ED, will be asked to participate. Once inclusion criteria are met (see “Inclusion and exclusion criteria” section), each patient will be randomly assigned to one of the multiple baseline conditions: 8, 12, or 15 evaluation days before the intervention. These multiple baseline conditions have been chosen following the current guides for single-case designs [34,38].

A random assignment will be carried out to reduce the threat of selection, and the fact of choosing three conditions is one of the established standards for this type of designs in order to reduce the threat of ambiguous temporal precedence and maturation [38]. Furthermore, the fact that all three conditions involve evaluation periods of 8, 12 and 15 days is due to the established standard of having a minimum of five data points in each phase to reduce the threat of attrition [38]. By choosing at least eight, we make sure to meet it even considering the probability that participants will forget to fill out the assessment one day. Actually, the reason for choosing those days is the temporal stability of the variables that we measured through the study.

In addition, the intervention will begin in a staggered manner with individual sessions (to reduce the threat of the history) and to reduce the threat of testing, a pre-treatment assessment will be performed prior to starting the baseline, to consider that the assessment process itself can have therapeutic effects on participants [38].

**Figure 1:**
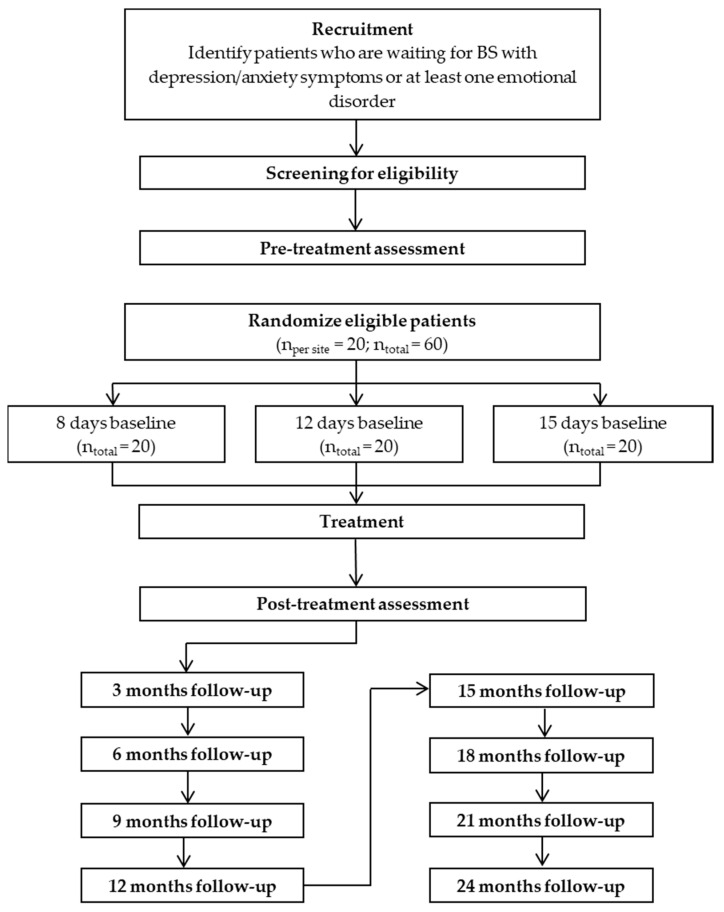
Study flow chart. BS: Bariatric surgery.

The study includes five assessment moments (pre-treatment, baseline, treatment, post-treatment, and follow-up, one every three months until two years after treatment completion). The flow chart of the study design is shown in Figure 1.

### 2.1. Sample and Recruitment

The study recruitment will start in September 2020 until December 2024. The study will be conducted in a public mental health center in Spain (Mental Health Unit of the Regional Hospital of Vinaròs, Castellón). Participants will be people over 18 years of age who have anxious or depressive symptoms, or at least one diagnosis of EDs according to Diagnostic and Statistical Manual of Mental Disorders five edition criteria [39] and who have been selected for a subsequent BS.

To calculate the sample size, we drew on a study that proposed stepwise rules of thumb for pilot studies based on the target effect size and the size of the future trial [40]. Previous works that study the efficacy of CBT procedures either in person or online have shown large variations in effect sizes, obtaining mainly medium to large effect sizes [41,42]. Taking these data into account, and expecting a 90% powered main trial and assuming a sample loss of 35% [43] we established a sample size of 20 participants per experimental condition for the present study, that is, a total sample of 60 participants.

### 2.2. Procedure

The participants who are referred to the mental health unit for having fulfilled the requirements to be candidates for BS, will be evaluated to see if they meet the rest of the inclusion criteria for this study. In Table 1, it can be seen the selection criteria used to choose the appropriate candidates to receive the BS. These criteria are based on the European Guidelines for Obesity Management in Adults, which indicates that a comprehensive obesity management can only be accomplished by a multidisciplinary obesity management team [44].

Participants’ evaluation and selection will be carried out by the clinical psychologists and psychiatrists of the mental health unit of the Regional Hospital of Vinaròs (Castellón). The clinical psychologists who will participate in this trial will be in charge of collecting all the information from the participants (alphanumeric codes assigned to the participants will be entered to safeguard their anonymity). The coded information will be given to the author J.O. to introduce it in the database and then, returned to the center.

Participants who have received the approval for BS by the multidisciplinary health team and also have met the inclusion criteria by the clinician (see “Eligibility criteria” section), will be invited to participate in the study through an informative document. They will also be provided with confidentiality and informed consent documents. After accepting to participate, an email will be sent to participants with a link through which they will be able to complete the pre-treatment evaluation on the Qualtrics survey platform [45]. Then, they will be informed by phone of the experimental condition to which they will have been randomly assigned, which may be 8, 12, or 15 days baseline assessment, and they will then complete online the baseline assessment protocol on the same platform [45]. Randomization to the different baselines will be done with randomizer software (www.randomizer.org). Randomization will be performed by a researcher unrelated to the study using the computer-generated sequence mentioned. In the program, the researcher will generate one set of 60 numbers, which will have a one to three range. Participants will be randomly assigned to the 8, 12 or 15 baseline days.

The baseline and psychological intervention will be conducted between the period of acceptance for BS and the BS implementation, that last approximately one year in public health settings. The intervention will be carried out in an online group format through the Cisco Webex platform. The UP will be applied in twelve weekly 2-h online group sessions. To comply with the experimental design of the multiple baseline, when each participant completes the baseline evaluation, they will receive the first online session in individual format (to receive the first session in stages), then they will continue with the second session and the rest of the treatment in group format. The content of each session is shown in Table 2. Participants will receive the therapy support manual [11]. The online group will consist of five to eight participants, one therapist (V.F.-G.), and one co-therapist (A.Q.-O.). For ethical reasons, if any of the patients feel uncomfortable during the study with the online group format, they may leave the group and receive individual onsite attention (treatment as usual). In this case, the content and estimated number of sessions will be the same, although the frequency will be stipulated by the mental health unit depending on its possibilities.

The study plans to conduct a follow-up assessment every three months until two years after treatment completion. All follow-ups will be conducted online. Considering the time interval of the baseline, the psychological intervention and the BS procedure, the two first follow-ups (three and six months after intervention) will be conducted before BS, and the rest will be conducted after BS. In addition, at the baseline and during treatment, the participants must fill out an online survey made up of 14 questions that will ask about the intensity of the emotions of happiness, sadness, anxiety, other emotions, difficulties in emotion regulation, body image, and emotional eating. Through this weekly evaluation, we expect to observe how the participants evolve in the different variables throughout the different treatment modules.

### 2.3. Eligibility Criteria

The inclusion criteria for participation in the project will be: (1) being over 18 years of age; (2) being a BS candidate; (3) presenting anxious or depressive symptomatology (moderate scores on the Beck Depression Inventory-II [46,47] and/or Beck Anxiety Inventory [48,49]) or meeting the criteria for at least one ED (anxiety, mood, and related disorders) on the International Neuropsychiatric Interview (MINI; [50]); (4) speaking Spanish or Catalan fluently; (5) committing to attend the sessions; (6) understanding and accepting the contents of the informed consent, expressed by signing it; (7) having Internet to fulfill the protocol assessments and to participate in the online intervention and (8) agreeing to maintain the prescribed medication (including dosage) if any, during the evaluation period and treatment. If medication stability is not possible, the participant’s data will be treated separately in the analyses.

The protocol also includes one exclusion criteria that may interfere: (1) having a severe condition that would require being prioritized for treatment, so that an interaction between the two interventions cannot be ruled out. These include a severe mental disorder (bipolar disorder, personality disorder, schizophrenia, or an organic mental disorder), suicide risk at the time of assessment, or substance use in the last three months (excluding cannabis, coffee, and/or nicotine).

### 2.4. Ethics

All participants who meet the inclusion criteria will sign the personal data protection document before randomization so that they have a notion of whom and for what purpose this study’s results will be used. Participants will be also informed of what the treatment consists of before starting it, as well as the duration and phases of the study (informed consent). Direct participation in the study will be voluntary. Participants will not obtain any financial or material compensation, and their participation will not imply any risk for them. The UP has already demonstrated its efficacy in previous experimental and quasi-experimental studies with different health problems (see “Introduction” section).

Data management will be carried out following the Spanish Royal Decree 1720/2008, of 19 January, which approves the Regulations for the development of the Organic Law 15/1999, of 13 December, on the protection of personal data [51]. The treatment, communication, and transfer of personal data will follow the provisions of the Declaration of Helsinki [52] in Law 14/2007 on biomedical research. As of 25 May 2018, the new legislation on personal data in the EU is fully applicable, specifically Regulation (EU) 2016/679 of the European Parliament and of the Council of 27 April 2016, on Data Protection (GDRP; General Data Protection Regulation). Under the aforementioned legislation, participants can exercise their rights of access, modification, opposition, and cancellation of data, for which they must contact their health professional of reference.

The data collected for the study will be identified by an alphanumeric code, and only the health professionals collaborating in the study will be able to relate these data to the participants and their corresponding medical records. Therefore, the identity of the participants in the study will not be revealed to any person, with exceptions in case of a medical emergency or legal requirement.

The study already has the approval by the Ethical Research Committee of the General University Hospital of Castellón (CEIm; CI/HIP version 1.0 de 20/07/2019). In addition, the protocol study has been registered at https://clinicaltrials.gov/ (6 March 2020; NCT04421443).

### 2.5. Measures

The evaluation protocol will be administered online by Qualtrics survey [45] at five different moments: pre-treatment, baseline, treatment, post-treatment, and follow-ups every three months until two years after the intervention. Assessment instruments will include demographic characteristics (age, sex, education, marital status, and employment status), a diagnostic interview, and well-established questionnaires for both primary and secondary outcomes. Next, the primary and secondary outcomes that will be evaluated at the pre, post-treatment, and follow-up periods will be described, and later the specific measures that will be used for the baseline evaluation and during the treatment will be specified. Table 3 shows the distribution of the measures that will be administered during the study.

#### 2.5.1. Primary Outcomes

Primary and secondary diagnosis according to the DSM-5 criteria will be evaluated with the International Neuropsychiatric Interview (MINI [50]). Subclinical symptoms of anxiety and depression will be evaluated through the Beck Depression Inventory-II (BDI-II [46,47]) and the Beck Anxiety Inventory (BAI [48,49]). The weight gain or loss of the participants will be evaluated through the Body Mass Index (BMI).

#### 2.5.2. Secondary Outcomes

Secondary outcomes can be grouped around: affectivity, personality traits, quality of life and interference, emotion regulation, aspects related to surgical complications, eating behaviors and body image, and satisfaction with and evaluation of the treatment received.

To assess affectivity, the Positive and Negative Affect Scale (PANAS [53,54]) will be administered to evaluate positive and negative affect. Personality will be measured with the NEO Five-Factor Inventory (NEO-FFI [55]), which offers a rapid and general measure of the Big Five personality traits of which we will only assess Neuroticism and Extraversion. The Quality of Life Index (QLI [56]) will be used to evaluate several aspects related to quality of life (i.e., physical disability, emotional well-being, self-care and independent functioning, occupational functioning, interpersonal functioning, social–emotional support, community and services support, personal fulfillment, spiritual fulfillment, and overall quality of life). EuroQol [57,58] is a generic instrument that will be used to measure health-related quality of life. It has five dimensions (mobility, personal care, daily activities, pain, and anxiety/depression), and a general state of health perceived through a visual analog scale. Similarly, the Maladjustment Inventory (MI [59]) will be used to evaluate the extent to which the subject’s current problems impact negatively on different areas of daily life, namely, work, social life, leisure time, relationship with the partner, family life, and overall adjustment in daily activities.

Regarding emotion regulation, it will be assessed using the Difficulties in Emotion Regulation Scale (DERS [60,61]), which presents five dysregulation dimensions: emotional lack of control, emotional rejection, life interference, lack of emotional attention, and emotional confusion. In addition, to assess the different emotion regulation skills that will be trained through the UP, we will use the Brief Experiential Avoidance Questionnaire (BEAQ [62,63]), which is a self-report questionnaire with 15 items that measure experiential avoidance; the Philadelphia Mindfulness Scale (PHLMS [64,65]), which is a 20-item questionnaire that assesses two mindfulness constructs: awareness and acceptance and the Emotion Regulation Questionnaire (ERQ [66,67]), which is a self-report questionnaire commonly used to assess two emotion-regulation strategies: cognitive reappraisal (six items) and expressive suppression (four items).

To assess surgical complications, the professional will be asked to refer the participant to the mental health unit a report describing the course of the operation and the recovery from it. Specific measures related to eating disorders will also be used, such as the Bulimic Investigatory Test (BITE [68,69]), which is a self-report questionnaire used to evaluate the presence and severity of bulimic symptomatology, and cognitive and emotional signs and symptoms associated with binge eating; the Body Satisfaction Questionnaire (BSQ [70,71]), which is a self-applied scale used to evaluate the fear of gaining weight, feelings of low self-esteem because of one’s appearance, the desire to lose weight, and body dissatisfaction, and Emotional Eating Scale (EES [72]), which is a 25-item self-report assessing a person’s tendency to cope with negative affect through eating. Participants’ weight will be checked monthly to calculate their body mass index (BMI).

Additionally, we created an ad hoc questionnaire to evaluate the participants’ evaluation of and satisfaction with the treatment received, the Satisfaction with Treatment Questionnaire (STQ). The Evaluation of the UP Components section consists of nine items that evaluate the extent to which the participants consider that the UP in general, and each of its components in particular, were useful to help them to regulate their emotions adaptively; the Satisfaction with Treatment section presents seven items that evaluate participants’ overall satisfaction with the treatment received. In both sections, higher scores show higher levels of positive evaluation and satisfaction. A total of 12 items will also be added to assess the participants’ opinion of the online evaluation through Qualtrics and of the Cisco Webex platform used to carry out the online evaluation. At the end of the questionnaire, six open-ended questions appear in which the participants can qualitatively express their opinion of different aspects of the treatment received and the delivery format.

All measures used in the study have been standardized in Spanish. Administration time is between 30 and 40 min for the MINI and approximately 90 min for the primary and secondary outcomes conjointly.

#### 2.5.3. Baseline Assessment

To facilitate the daily baseline assessment, we have summarized in one item the variable we want to evaluate, for example, the emotion regulation strategies. For this purpose, we have chosen the item with the greatest factor load for each variable [73]. Specifically, participants will answer a battery of 19 questions: six questions about the presence and intensity of specific emotions (e.g., happiness, sadness, anxiety); five questions to assess the five subscales of the Difficulties in Emotion Regulation Scale (DERS [60,61]), which are emotional lack of control, emotional rejection, life interference, lack of emotional attention, and emotional confusion (e.g., “During this day, to what extent have you paid attention to your feelings?”); two questions that refer to the two subscales of the Emotion Regulation Questionnaire (ERQ [66,67]), which are reappraisal and suppression (e.g.,” During this day, to what extent have you been able to control your emotions by changing the way you think about the situation you were in?”); two questions that refer to the two subscales of the Philadelphia Mindfulness Scale (PHLMS [64,65]), which are acceptance and awareness (e.g., “During this day, when your emotions have changed, to what extent have you been aware of it immediately?”); one question to assess experiential avoidance based on the Brief Experiential Avoidance Questionnaire (BEAQ [62,63]) (“During this day, would you say that one of your biggest goals has been to be free of any painful emotion?”); one question from the Emotional Eating Scale (EES [72]) regarding the impulse to eat as a consequence of having experienced an intense emotion (“During this day, to what extent have you felt the urge to eat as a result of experiencing intense emotions?”) and one last question from the Body Satisfaction Questionnaire (BSQ [70,71]), which asks about the extent to which the participant has been satisfied with their body image (“During this day, how satisfied have you been with your body image?”).

#### 2.5.4. Assessment during Treatment

The assessment during treatment battery involves continuing to fill in the same baseline questions once a week. In addition, before starting each online session, participants will be asked to fill in an emotional scale to assess its presence, intensity, and interference during the last week. Participants can choose the emotion or emotions that should be assessed. The UP offers four different emotional rating scales: The Overall Anxiety and Depression Severity and Impairment Scales (OASIS; ODSIS [74,75,76]), Other Emotions Severity and Impairment Scale (e.g., guilt, shame, or anger) (OESIS [77]), and a Positive Emotion Severity and Impairment Scale (e.g., happiness) (PESIS [77]; Supplementary Materials). All of them are used in the UP to help patients to continuously monitor the scores throughout the sessions and their progress over the treatment.

### 2.6. Data Analysis

The analyses will be carried out with the statistical package IBM SPSS Statistics version 22.0 for Windows [78]. First, the sociodemographic characteristics of the sample will be analyzed with descriptive statistics, calculating the mean and standard deviation of the scores in the different questionnaires administered. Next, a missing-value analysis and the Little Missing Completely At Random (MCAR) test will be performed to determine whether or not the distribution of missing values is random, and therefore whether the last observation made (LOCF) can be used. Internal consistency will be explored using Cronbach’s alpha.

As the sample size is expected to be less than 50, the normal distribution of the variables will be verified with the Shapiro–Wilk normality test. Depending on the result of this test, parametric or non-parametric repeated measures analysis will be carried out to verify whether or not the differences in the scores of the variables measured at different times are statistically significant. In case the variables follow a normal distribution, parametric repeated measures analysis will be carried out, specifically the Multivariate Analysis of Variance (ANOVA), and in case the variables do not follow a normal distribution, non-parametric repeated measures analysis will be carried out, specifically the Friedman test. If the repeated measures analysis show statistically significant differences between the evaluation time points, post-hoc comparisons will be carried out to correct the level of significance to avoid increasing the type I error. Thus, regarding adjustment for multiple comparisons, the Bonferroni correction will be carried out in case of having performed parametric analysis, and the Wilcoxon signed rank test in case of having performed non-parametric analysis in the comparison of means. More detailed information on this has been added in the data analysis section, explaining step by step which statistical analyzes will be carried out.

Finally, the Reliable Change Index (RCI), which assesses the clinically significant change obtained to determine in which variables the scores have approached those of the normative sample, will be calculated. A clinically significant change will be considered if the RCI score obtained is equal to or greater than 1.96 [79].

Another aspect that we consider important to analyze is how the participants change their scores in each of the variables evaluated based on the content addressed each module. For this purpose, a visual analysis of the changes in the scores will be carried out, to see how the slopes change in the different phases of the study (evaluation and treatment), and in the different modules within the treatment. This visual analysis has been used in previous studies [21]. To carry out this visual analysis, the data from 19 questions that the participants will fill in weekly will be used.

Attendance at sessions will also be recorded to calculate the attendance rate, which will be used as an indicator of viability and acceptance of treatment by users. For the same purpose, quantitative and qualitative analyses will be carried out. At the quantitative level, the participants’ evaluation of and satisfaction with the treatment received will be evaluated using an ad hoc designed questionnaire (see “Measures” section). Qualitatively, participants will be asked to answer six main questions about treatment, and their answers will be analyzed by selecting and classifying the information through a segmentation process, identifying key themes and categories of analysis [80].

## 3. Expected Results

Based on the reviewed bibliography, the type of design of the present study, the proposed objectives and the characteristics of the intervention that will be carried out, we hope that the results will reveal the feasibility and clinical usefulness of the UP applied in an online group format, in a mental health setting of the national health system for candidates of BS who have at least one diagnosis of ED or emotional symptoms. The concrete results that are expected to reach this conclusion are shown in more detail in Table 4.

Reporting of results will follow the Consolidated Standards of Reporting Trials (CONSORT) recommendations, specifically its extension for designs N-of-one Trials [81].

## 4. Discussion

Obesity and being overweight have become a serious public health problem due to their high prevalence and associated costs [2,9]. EDs are highly present in people with obesity [7], and this can interfere with a commitment to voluntary weight loss [82] and it is also associated with worse post-intervention outcomes [11]. An intervention based on emotion-regulation training could help those patients to achieve both aims, to lose weight before the BS to prevent surgery problems and the emergence of emotional symptoms after the BS, maintaining their emotional and physical achievements over time. In addition to the clinical implications, the results derived from this study may also have an important economic impact. Thus, health policies and the managing of this health condition in public health settings could be also influenced.

To prove this, longitudinal studies are needed and some limitations regarding the interventions (specific CBT versus transdiagnostic intervention) and the delivery formats (onsite versus online) must be considered. The use of the UP in an online group intervention format in a public mental health setting with people waiting for BS has the following benefits:

1. It can allow clinicians to use a single treatment for those candidates who present different EDs, with comorbidity, and also with subclinical symptoms or unspecified disorders [15].
2. In other public health systems similar to the Spanish one, where onsite specific CBT is the most common delivery format, and therapy sessions occur at long intervals (e.g., more than a month) due to waiting lists, a UP online group intervention can help to increase the frequency of sessions and reduce costs because there are more patients treated simultaneously (five to eight patients in the same group), which can facilitate better patient care.
3. Thanks to the online format, this intervention will facilitate access to psychological interventions in those candidates who face mobility challenges due to their obesity condition [31]. This innovative approach would be in line with the goals of the World Health Organization proposed in the mental health action plan to use electronic technologies to expand the delivery of mental health care [83].
4. The fact of receiving quarterly follow-ups up to two years after treatment allows guaranteeing the prevention of relapses and maintaining the results, especially beyond the year and a half or two years, which is the moment in which the literature recognizes that pre-BS problems tend to reappear [42].
5. Furthermore, this advantage offered by long term relapse prevention follow-ups leads to a condition of reduced healthcare costs that are associated with the care of comorbid health problems in this type of patients [3].
6. And finally, it is known that group therapy provides benefits to the patient that are not obtained with individual treatment, such as reducing isolation, facilitating social support, and learning from the experiences of others [33], which could improve its efficiency.

Our study also has some limitations. First, some people who are going to undergo BS will prefer individual treatment, which could be a barrier to enroll those participants in our study (e.g., abandonment or decrease in UP satisfaction and effectiveness). In this case, it could be explained to the patient that group treatment resulted in greater weight losses than individual treatment, even for those clients with a preference for individual therapy and that matching clients with their preferences for individual or group therapy did not enhance treatment outcome either in terms of weight loss or improvements in psychological functioning [84]. Furthermore, a study in a sample of people with EDs diagnosis attended to in public mental health settings in Spain found that the majority of participants preferred receiving psychological treatment in an individual format, followed by group format, and, rarely, in an online format, so it will be necessary to explain to patients the advantages and disadvantages of receiving psychotherapy through individual, group, or online format to help them to decide whether or not to participate in this study [85]. In this sense, the arguments that must be strengthened to justify a group application, in addition to those mentioned previously, are the possibility of sharing experiences, the opportunity to learn from others and receive comments and support and for the online format, convenience [85]. The second main limitation, is that, due to this being a pilot study, the results must be interpreted with caution, as further RCT studies with greater rigor will be required. In this sense, it should be noted that this kind of study is an advisable cost-effective method to preliminarily examine the efficacy, feasibility and/or implementation of recent intervention programs or applications with different samples [86].

Despite the aforementioned limitations, the present study may have different implications. At the research level, it will be the first pilot study aimed at evaluating the feasibility and clinical utility of an online group format of a transdiagnostic intervention for the treatment of ED in public settings in Spain with people waiting for BS, allowing us to increase the evidence on UP’s effectiveness, flexibility, and versatility. At a clinical level, the results of the study will reveal whether UP can improve emotional symptoms in candidates and, therefore, improve the effects of BS in the long term. This would have important implications for patients, as it could achieve much more notable improvements after the intervention, and would serve to prevent relapses that generally appear in the long term around two years after BS.

## 5. Conclusions

In sum, the present study supports the idea of the need to test new forms of psychological interventions with patients waiting for BS [12]. This would allow us to improve the previous psychopathology, and, therefore, to potentiate the effects of BS in the short and long term [2].

The UP may be a good treatment option, considering that it is directed at EDs (which are the most prevalent in people with obesity problems) [12], and that it allows addressing comorbidity, subclinical symptoms, and unspecified disorders [15].

In addition, its application through an online group format can provide extra advantages, as it allows access to treatment for people with mobility problems, something very common among candidates for BS [31], and also fosters social support among the members of the group [33].

Based on all this, the results of the development of this study may have important implications for the National Health System, which will be able to meet the psychological needs of patients with obesity problems, improving their quality of life before the intervention, and enhancing its results in the long term, which will mean a reduction in costs to the system and more specialized and multidisciplinary care for patients who are in this situation.

## Figures and Tables

**Table 1:** Inclusion criteria and main contraindications for BS.

**Inclusion Criteria**
- BMI >40 or 35 with associated major comorbidities
- Age between 18 and 65 years
- Long-standing obesity (3–5 years)
- Failure in dietary attempts and treatments under control
- Absence of anesthetic contraindication and acceptable surgical risk
- Not having endocrine causes of obesity
- Understanding of the weight loss process, associated problems and the stated objective
- Commitment to adhere to the monitoring standards
**Contraindications**
Relative
- Clearly unfavorable family environment
- Personality disorder
- Psychogenic vomiting
- Hyperphagia in other psychological disorders or other eating disorders
- Mild intellectual disability
- Psychotic disorders without positive symptoms
- Any psychiatric illness that significantly hinders the good follow-up and completion of the guidelines and medical indications for this process and may worsen the patient’s state of health
Absolute
- Drug dependency
- Moderate or severe intellectual disability
- Psychotic disorder with positive symptoms
- Bulimia nervosa

**Table 2:** Treatment content split by session.

Session Number	Content
Session 1	Setting Goals and Maintaining Motivation
Session 2	Understanding your Emotions: What is an Emotion?
Session 3	Understanding your Emotions: Following the ARC (Antecedent, Response, Consequence)
Session 4	Mindful Emotion Awareness—I
Session 5	Mindful Emotion Awareness—II
Session 6	Cognitive Flexibility—I
Session 7	Cognitive Flexibility—II
Session 8	Countering Emotional Behaviors
Session 9	Understanding and Confronting Physical Sensations
Session 10	Putting it into Practice: Emotion Exposures—I
Session 11	Putting it into Practice: Emotion Exposures—II
Session 12	Recognizing Accomplishments and Looking to the Future

**Table 3:** Distribution of the variables administered throughout the intervention.

Measures	Pre-Treatment	Baseline	Treatment	Post-Treatment	Follow-Ups ^1^
Demographic characteristics	X				
MINI	X			X	X
BDI-II	X			X	X
BAI	X			X	X
BMI	X	X	X	X	X
PANAS	X			X	X
NEO-FFI	X			X	X
QLI	X			X	X
EuroQol	X			X	X
MI	X			X	X
DERS	X			X	X
BEAQ	X			X	X
PHLMS	X			X	X
ERQ	X			X	X
Surgical Complications	X				
BITE	X			X	X
BSQ	X			X	X
EES	X			X	X
OASIS			X		
ODSIS			X		
OESIS			X		
PESIS			X		
STQ				X	X
Baseline assessment battery ^2^		X			
Assessment battery during treatment ^3^			X		

Note: MINI: International Neuropsychiatric Interview; BDI-II: Beck Depression Inventory-II; BAI: Beck Anxiety Inventory; BMI: Body Mass Index; PANAS: Positive and Negative Affect Scale; NEO-FFI: NEO Five-Factor Inventory; QLI: Quality of Life Index; MI: Maladjustment Inventory; DERS: Difficulties in Emotion Regulation Scale; BEAQ: Brief Experiential Avoidance Questionnaire; PHLMS: Philadelphia Mindfulness Scale; ERQ: Emotion Regulation Questionnaire; BITE: Bulimic Investigatory Test; BSQ: Body Satisfaction Questionnaire; EES: Emotional Eating Scale; OASIS: Overall Anxiety Severity and Impairment Scales; ODSIS: Overall Depression Severity and Impairment Scales; OESIS: Other Emotions Severity and Impairment Scale; PESIS: Positive Emotion Severity and Impairment Scale; STQ: Satisfaction with Treatment Questionnaire. ^1^ Quarterly follow-ups up to two years after treatment; ^2^ For more information about the specific items that make up the baseline assessment battery see section “Baseline assessment”; ^3^ For more information about the specific items that make up the assessment during treatment battery see section “Assessment during treatment”.

**Table 4:** Main expected outcomes. UP: Unified Protocol for the transdiagnostic Treatment of Eds.

**Clinical Usefulness of the UP for Bariatric Surgery Candidates**
**Group Results:**
- Depending on the characteristics of the sample obtained (we will check if the parametric assumptions are met), the results of the parametric/non-parametric tests that allow comparing the group scores between the different time points are expected to show statistically significant differences in the desired direction.
**Individual Results:**
- To obtains statistically significant differences (Reliable Change Index; RCI) between the scores obtained at the pre-treatment, baseline, and post-treatment on the primary and secondary measures.
- A maintaining of improvements obtained after the application of the UP in the long term (follow-ups of up to two years), with statistically significant differences (Reliable Change Index; RCI) between the scores obtained at the pre-treatment/post-treatment and follow-ups on the primary and secondary measures.
**In both cases, the expected directions for the different variables are:**
- An increase for the variables positive affect, extraversion, quality of life and health-related quality of life.
- A decrease for the variables negative affect, neuroticism, interference of symptoms, emotional dysregulation, bulimic symptomatology, dissatisfaction with body image and emotional eating.
Feasibility of the UP for Bariatric Surgery Candidates
- High session attendance rates.
- High scores on the STQ both in the questions that refer to the treatment itself and its different modules, and in the questions that refer to the group and online format.

## Supplementary Materials

Unified Protocol for Transdiagnostic Treatment of Emotional Disorders (therapist guide and workbook) are available at https://www.oxfordclinicalpsych.com/view/10.1093/med-psych/9780190685973.001.0001/med-9780190685973; https://www.oxfordclinicalpsych.com/view/10.1093/med-psych/9780190686017.001.0001/med-9780190686017. Both Spanish versions are available at https://www.alianzaeditorial.es/libro/manuales/protocolo-unificado-para-el-tratamiento-transdiagnostico-de-los-trastornos-emocionales-manual-del-terapeuta-david-h-barlow-9788491814795/; https://www.alianzaeditorial.es/libro/manuales/protocolo-unificado-para-el-tratamiento-transdiagnostico-de-los-trastornos-emocionales-manual-del-paciente-david-h-barlow-9788491814818/.
